# A large-scale immuno-epidemiological simulation of influenza A epidemics

**DOI:** 10.1186/1471-2458-14-1019

**Published:** 2014-09-29

**Authors:** Sarah Lukens, Jay DePasse, Roni Rosenfeld, Elodie Ghedin, Ericka Mochan, Shawn T Brown, John Grefenstette, Donald S Burke, David Swigon, Gilles Clermont

**Affiliations:** Department of Mathematics, University of Pittsburgh, Pittsburgh, PA USA; Pittsburgh Supercomputing Center, Carnegie Mellon University, Pittsburgh, PA USA; School of Computer Science, Carnegie Mellon University, Pittsburgh, PA USA; Center for Vaccine Research, Department of Computational and Systems Biology, University of Pittsburgh, Pittsburgh, PA USA; Department of Critical Care Medicine, University of Pittsburgh, Pittsburgh, PA USA; Department of Biostatistics, University of Pittsburgh Graduate School of Public Health, Pittsburgh, PA USA; Department of Computational and Systems Biology, University of Pittsburgh, Pittsburgh, PA USA; Department of Epidemiology, University of Pittsburgh Graduate School of Public Health, Pittsburgh, PA USA; Department of Biological Sciences, University of Notre Dame, South Bend, IN USA

## Abstract

**Background:**

Agent based models (ABM) are useful to explore population-level scenarios of disease spread and containment, but typically characterize infected individuals using simplified models of infection and symptoms dynamics. Adding more realistic models of individual infections and symptoms may help to create more realistic population level epidemic dynamics.

**Methods:**

Using an equation-based, host-level mathematical model of influenza A virus infection, we develop a function that expresses the dependence of infectivity and symptoms of an infected individual on initial viral load, age, and viral strain phenotype. We incorporate this response function in a population-scale agent-based model of influenza A epidemic to create a hybrid multiscale modeling framework that reflects both population dynamics and individualized host response to infection.

**Results:**

At the host level, we estimate parameter ranges using experimental data of H1N1 viral titers and symptoms measured in humans. By linearization of symptoms responses of the host-level model we obtain a map of the parameters of the model that characterizes clinical phenotypes of influenza infection and immune response variability over the population. At the population-level model, we analyze the effect of individualizing viral response in agent-based model by simulating epidemics across Allegheny County, Pennsylvania under both age-specific and age-independent severity assumptions.

**Conclusions:**

We present a framework for multi-scale simulations of influenza epidemics that enables the study of population-level effects of individual differences in infections and symptoms, with minimal additional computational cost compared to the existing population-level simulations.

**Electronic supplementary material:**

The online version of this article (doi:10.1186/1471-2458-14-1019) contains supplementary material, which is available to authorized users.

## Background

Models of infectious disease epidemics, such as due to influenza A virus, have proven useful for understanding dynamics of disease transmission, vaccination strategies, and are frequently used as a decision support tool by public health officials. Single scale models of influenza A virus have been developed both on the host-level and on the population level in order to study epidemiological, immune response and viral characteristics of influenza A infection [1].

On the population scale, continuous models based on SIR (susceptible/infected/removed) approaches have been used for large-scale epidemiological predictions [2]. Continuous SIR models assume homogeneous populations and any model assumption about heterogeneity (such as human characteristics, network structure, and environmental factors) increases the number of equations and parameters, necessitating changing the model structure for any additional assumption. An alternative discrete approach to the study of disease epidemics is through agent-based models (ABM), where individuals are represented as autonomous agents whose infectious status is followed in time. Population density, age-structure, travel patterns and inter-individual contact patterns are derived or inferred from available census and demographic data. The infection propagates in the population according to disease transmission and duration rules, where inter-individual variability is represented in the form of statistical distributions. Because ABMs evolve individual agents, simulations require considerable computational cost. Many large-scale collaborative networks have developed and use large platform ABMs to make epidemiological predictions [3–6] and the impact of a variety of mitigation strategies. ABMs have been used in studies of school closure strategies [7, 8], in determining the role of subway travel in an epidemic [9], in the role of presenteeism on disease transmission in the workplace [10], and in vaccine allocation [11, 12].

Immuno-epidemiological models explore how host-level immune response affects population-level epidemiological patterns [13] and allow much finer tuning of host-behavior, compared to population-level models. The nested modeling approach embeds a mechanistic model of host-pathogen disease dynamics into an epidemiological model of infection by linking epidemiological parameters such as transmission rate or duration of infectiousness [14–16]. Immuno-epidemiological models consider assumptions about acquired immunity to study spread of infection [17–19] or host-parasite co-evolution [14, 20]. To account for heterogeneity in immune response, SIR models may be refined to incorporate individual host response through different linking mechanisms. Understanding the biological mechanisms underlying these variations in host response is important for estimating disease parameters and for designing optimal prevention strategies [21, 22].

Symptoms of influenza infection include fever, sore throat, chills, and cough, and have been attributed to the immune response to viral infection [23–26]. Clinical markers related to disease and disease-severity, largely due to host-immunity factors and prior exposures, are referred to as clinical phenotypes. Mathematical models of within-host influenza A infection have been used in estimation of kinematic parameters such as infection and clearance rates of the virus, as well as in prediction of dynamics and drug therapy strategies for individual infection [27, 28], yet have not been used to provide a mechanistic basis for varying disease phenotypes. Immuno-epidemiological modeling could be used to account for variability in clinical phenotype and evaluate the impact of symptom-based, biologically-based, and potentially behavior-based mitigation strategies at the population level.

A key challenge in deriving a realistic description of within-host responses is lack of data at the host-level. Human data is available from volunteer challenge studies where volunteers are inoculated intranasally, tend to be young (ages 18-35), healthy, and pre-screened for existing immunity to the experimental IAV strain. For H3N2 it has been shown that illness following intranasal inoculation is milder than illness following aerosol inoculation, manifested by shorter duration of cough and fever [29]. Mechanisms of transmission of naturally acquired infection include contact transmission, droplet spray transmission, and aerosol transmission. Therefore, using a host-level model to predict individual response to influenza infection requires altering model parameters based on healthy volunteer data in a biologically meaningful way.

Morbidity and mortality resulting from influenza infection is highest among the elderly (>65) and children. In a study of pandemic H1N1 in Ontario Canada, recovery was faster among patients under 18 years of age [30]. To gain qualitative insight of how host-level dynamics are varied as a function of age, animal studies prove to be useful. A study between aged and adult mice infected with sublethal doses of influenza virus (A/Puerto Rico/8/1934) showed slower recovery and delay in immune system activation and virus clearance in aged mice [31]. Differences in mouse response as a function of age provide insight in how to vary baseline responses in a within-host model across different age groups, while synthetic population data sets provide reasonable age distributions across a population [32, 33].

Naive integration of a within-host model in a populationlevel ABM requires the evaluation of a nonlinear system of differential equations for each individual infected, which is computationally prohibitive for realistically large simulations. In this manuscript, we present a prototypical hybrid immuno-epidemiological ABM model linking an equation-based within-host model and providing mechanistically-based host variability to an agent-based population level model. For outputs of the within-host model we choose the infectivity and symptoms scores during the time course of infection, which reflect likelihood of transmission and stay-at-home behavior of an infected individual. In this fashion, we can replicate existing results from simulated epidemics, but we introduce additional flexibility in studying variation of host-response at no additional computational cost. To eliminate computational cost, we replace the exact response with an approximation obtained by analyzing the most significant response modes of the within-host model. Further, the within-host model is simple to program and implement, and can be used to represent within-host dynamics in a variety of population-level platforms. We apply the hybrid ABM model to study questions arising from individual heterogeneity that cannot be addressed using a single scale model. In particular, we examine the role of varying severity of disease as a function of age on the dynamics of simulated epidemics. We find that epidemiological estimates such as attack rate and incidence are reduced when differences in host response across age groups are considered. Use of the immuno-epidemiological ABM model opens up many possibilities regarding the realistic impact of influenza on populations with different contact patterns or on mitigation strategies which depend on host phenotype or behavior.

## Methods

### Human volunteer data

The data set consists of viral load and symptom data from five previously published human volunteer studies conducted between 1995 and 1999 for 84 total individuals infected with A/Texas/36/91 (H1N1) [23, 24, 34–36]. Of these studies, only one [35] (n =17) does not contain symptom data. The viral titers across the five studies are all reported as averages across individuals in the respective studies in units of *log*_10_ TCID _50_/mL. To adjust the data across studies, we used mixed-effects modeling to compute a vertical shift for each study (random-effect), assuming that the distance between each curve is minimized. We computed weighted means and standard deviations (fixed-effect) on the shifted data.

In all studies, symptom scores were measured twice a day, and averaged. Across studies, 65 of the 67 individuals with symptoms data experienced at least one symptom. While similar symptoms were scored, their measurements were on different scales across studies. As done in [37], we scaled each study curve to its maximum clinical score. The summary curves for both titer and symptoms were calculated as a weighted average with weights as the number of individuals considered in each study. Data is summarized in the Additional file 1.

### Overview of the immuno-epidemiological ABM model

**Figure 1:**
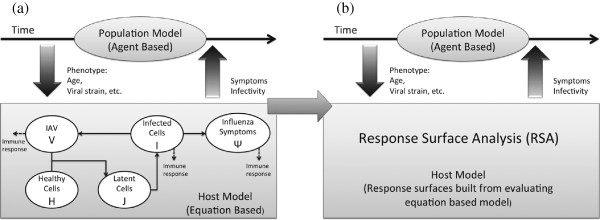
**Schematic diagram of the hybrid model.** **(a)** Naive combination of equation based model of virus and symptoms dynamics of influenza A infection with a population level model. Clinical phenotypes of infection are input into the host-level model, which is integrated numerically and returns symptoms and infectivity of an infected individual to use for simulation of an epidemic. **(b)** In the hybrid ABM model response surface replaces host-level ODE model.

We describe the process in which we link an equation-based model of within-host influenza A dynamics to a population scale ABM. A diagram of the procedure is shown in Figure 1. A collection of agents representing the population interacts within the simulation environment, transmitting infection in the process. Knowledge of the within-host immune response is used to determine the response of each agent to infection. Within each host, the immune response is reduced using principal component analysis (PCA) to identify significant directions in parameter space that describe most of the variability in immune response. Response surface analysis (RSA) captures the relationship between input (now simplified to two principal components) and model response (infectivity and symptoms) as an algebraic equation. Randomization over the principal components is used to generate population response variability. With this framework in place, we simulate disease outbreaks to see how changes at the within-host level affect a simulated epidemic in Allegheny County, PA.

### Estimation of parameters for virus/symptoms model

We enhanced a target-cell limited ordinary differential equation (ODE) model first proposed by Baccam *et al.* [38] to model viral and symptoms dynamics within a host with an additional term to incorporate symptoms dynamics:
12345

A schematic of the model is shown in the left panel of Figure 1(a). An individual is infected by an initial viral load *V*(0)=*V*_0_, which infects target cells (*H*) of the respiratory tract with infection rate *β*. Virus replicates within latent phase cells (*J*), which after average rate of time *k* become infectious (*I*) and shed virus at a rate *p*. Infected cells die at rate *δ*, primarily through cell necrosis, which triggers productions of cytokines such as type I interferons (IFN), interleukin(IL)-6, tumor necrosis factor (TNF)- *α* and IL-1 [39]. The novel contribution of this model is the inclusion of a variable *Ψ* quantifying the intensity of systemic symptoms of influenza, such as malaise and fever. Of all the variables of the model, it is most reasonable to have systemic symptoms dependent on infected cells, since both flu-like symptoms and severity of systemic symptoms have been shown to be correlated to cytokine levels [40, 41], particularly IL-6 and IFN- *α*/*β* [23, 24, 42], and inflammatory cytokines are, in turn, produced by infected cells. It has also been noticed experimentally that the number of days of viral shedding is correlated with cytokine levels, severity of symptoms, and mucus weight [43, 44]. While resolution of symptoms is a complex process, we assume a constant decay rate *a*. Model trajectories include viral load and symptoms scores, which are used as determinants of infectivity and symptomaticity in the population level simulation and represent state variables corresponding to data. We use Bayesian inference to construct an ensemble of models consistent with the data in order to explore the parameter space associated with our model and data. We sample the Bayesian posterior density *P*(***α***|*D*) using a Markov Chain Monte Carlo (MCMC) method with parallel tempering [45–48]. Ensemble modeling results in a collection of trajectories consistent with the data, in which each trajectory corresponds to a parameter set from the posterior distribution.

### Abstraction of the within-host model for population-level evaluation

The ensemble of model parameters ***α*** sampled from the posterior distribution *P*(***α***|*D*) is used to determine how the immune response depends on model parameters, and how the response varies over the population [49, 50]. As large ranges of parameter values may all fit the data, we seek to identify directions in parameter space in which the model behavior is tightly constrained in order to make predictions. The ensemble of parameters can be characterized using the covariance matrix *Θ*=*cov*(***α***), or the Hessian matrix of the cost manifold at a global minimum in parameter space; these two matrices are related as *H*≈*Θ*^-1^. A singular value decomposition, or PCA, of this approximated Hessian provides principal directions in parameter space corresponding to maximum changes in the model with respect to the cost function, here represented by total deviance from empirical virus and symptom values at each day during infection. We use the principal directions to vary parameter values for model evaluation, and define the hyper-plane *P*=*span*{*d*_1_,*d*_2_} as the plane in parameter space spanned by the two leading principal directions. The computational domain is a discretization of the box [-0.5,0.5]× [-0.5,0.5]⊂*P* into a 20 by 20 grid. We select a random sub-sample of 1,000 parameter sets from the ensemble, which characterizes the “origin” of the hyper-plane. For each point on the grid, parameters are deviated from baseline along two principal directions by a distance specified by the grid coordinates. The deviated values are simulated in the model (Equations 1-5) yielding viral loads and symptoms responses for 11 days. The simulated median surface is then parameterized by a quadratic polynomial, which has the form
6

### Population level model

The within-host representation is linked to the Framework for Replication of Epidemiological Dynamics (FRED) ABM software, an open source population-level large scale modeling system developed by the University of Pittsburgh Models of infectious Disease Agents Study (MIDAS) Center of Excellence. We use data from the synthetic population database, which is freely available and based on 2005-2009 U.S. census data [32] from Allegheny County, Pennsylvania. The data from the 1,164,879 agents assigns characteristics and behaviors such as occupation and workplace or school [32, 33] to each individual. Each household is assigned a latitude/longitude coordinate, income, size, and the sex, race, and age of household occupants, representing the distribution of U.S. households. Behavior and daily routine of agents are based on a set of assumptions that describe contact patterns and movements of individuals. Individuals interact daily with schools or workplaces, household, and neighborhood networks. In each scenario, individuals contact infection based on a fixed mean number of people per day. For detailed descriptions on how agents interact and perform daily routines, the reader is referred to [4, 7, 9–12, 51].

At any time in FRED, each agent is in one of four states: susceptible (S), exposed (E), infectious (I) and recovered (R). We assume that individuals transition from the exposed state to the infectious state when the computed infectivity crosses a defined threshold. All agents are initially susceptible to infection, and at *t*=0 days, 100 random agents are initialized in the exposed state. Contact with an infectious person has an assigned probability of transmission, which is further modulated by the individual’s infectivity (See Additional file 1).

## Results

### Model ensemble

**Figure 2:**
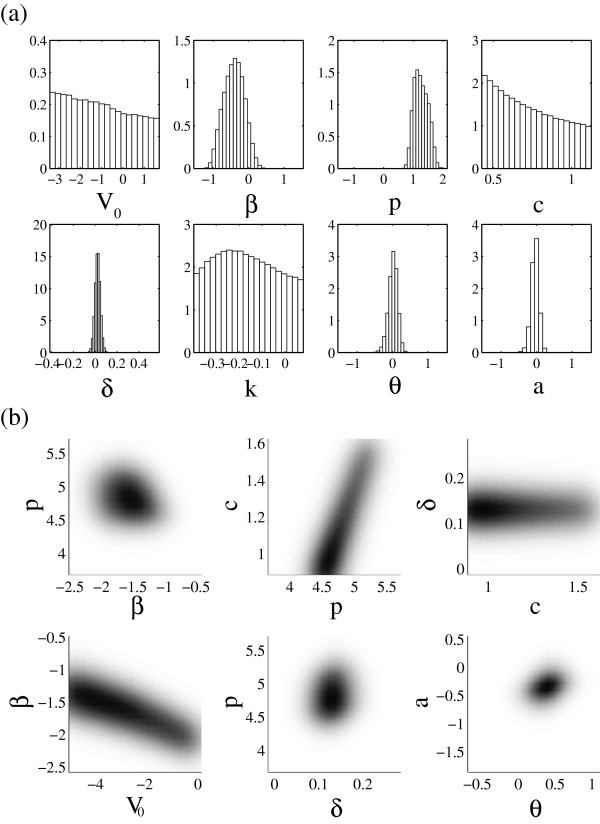
**Parameter distributions and correlations of model parameters.** **(a)** One dimensional projections (marginal distributions) of posterior distribution along each (log) parameter axis. Biological bounds constrain values of *V*_0_, *c*, *δ* and *k*. Other parameters are normalized against their baseline value and shown in log scales. **(b)** Density plots of correlations of select parameter pairs of parameters.

The marginals and correlation plots for the posterior distribution *P*(***α***|*D*) are shown in Figure 2 for a sample of 1 million parameter values. Values for the best-fit parameter set are provided in Table 1. Calculation of correlation coefficients across the ensemble shows three parameter pairs to be significantly correlated. Viral production (*p*) and degradation rates (*c*) are highly correlated (with correlation coefficient *R*=0.90), as are symptoms onset (*θ*) and clearance (*a*) terms (*R*=-0.85). A parameter pair of interest are inoculum size (*V*_0_) and infection rate (*β*), with correlation *R*=-0.80. To fit the data, either a small initial viral load pairs with a fast infection rate for the viral trajectory to reach the first data point, or a larger initial viral load pairs with a slower infection rate.

**Table 1:** **Parameter values**

	***V***_0_	***β***	***p***	***c***	***δ***	***k***	***θ***	***a***
Description	Inoculum	Infection	Viral	Viral	Infected	Latent	Rate of	Symptoms
	size	rate	production	clearance	cell decay	cell	symptoms	clearance
			rate	rate	rate	rate	onset	rate
Units	TCID _50_ mL	(TCID mL day ^-1^)	TCID mL day ^-1^	(day ^-1^)	(day ^-1^)	(day ^-1^)	day ^-1^[*S*]^-1^	(day ^-1^)
Baseline (***α***_0_)	0.02	0.083	4000	3	1.3	5	2.5	0.55
Lower Bounds	0.75e-5	*β*_0_/35	*p*_0_/35	8	0.5	2	*θ*_0_/35	*a*_0_/35
Upper Bounds	1	35 *β*_0_	88 *p*_0_	40	2	6	35 *θ*_0_	35 *a*_0_
Best fit	7.50e-6	0.0674	40356	8.00	1.364	3.684	2.75	0.498

The reported bounds are used for parameter estimation. Epithelial cells are scaled and hence have non-dimensional units. Symptoms are based on a symptoms score value, which unit we denote by [*S*].

**Figure 3:**
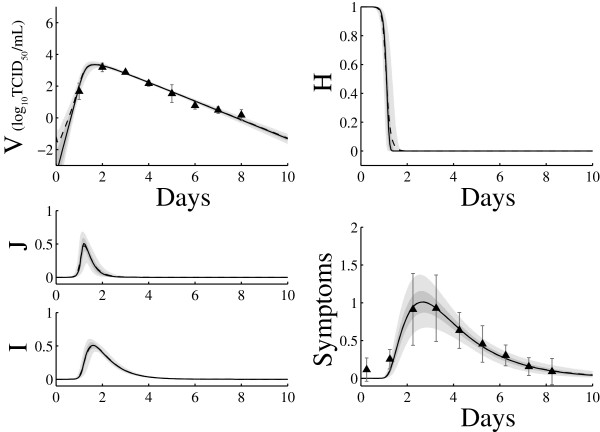
**Ensemble model trajectories with median (black), 25-75 quartile ranges (dark gray) and 5-95 quartile ranges (light gray) across a random sub-sample of 15,000 parameter sets from the posterior distribution.**

Using a random sub-sample of 15,000 parameter sets from the ensemble, statistical representation of trajectories showing median, 25-75, and 5-95 percentiles of variable values are plotted at each time point in Figure 3. The model fits data well, including the variability in the symptom response. There is variability across trajectories in viral load during the first 2 days, due to the different inoculum sizes *V*_0_ spanning several log scales and related to the correlation between *V*_0_ and the infection rate.

### Representing biological phenotypes

Singular value decomposition on the approximated Hessian of the posterior distribution was performed to identify candidate directions. Due to the strong correlation of the inoculum size *V*_0_ with the infection rate *β* revealed in analysis on the ensemble, it is only necessary to vary one of these two parameters (*V*_0_ and *β*) to achieve observable differences and the model is more sensitive to changes in infection rate. Therefore, we fix *V*_0_ associated with a given parameter set and reduced the parameter space to seven dimensions. Variation in inoculum size is still present due to the range of values sampled in the posterior distribution. Combined, directions one and two account for 89.2% of the variability, while the other five components account for the remaining. The leading two components give the following directions in parameter space:
78

**Figure 4:**
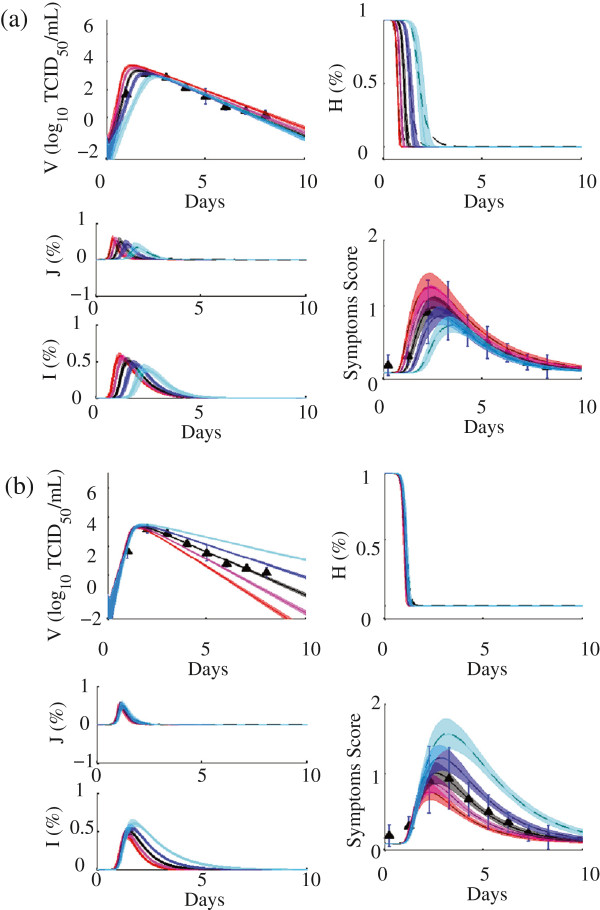
**Variability of host-level model responses along principal directions of the ensemble.** **(a)** *d*_1_ and **(b)** *d*_2_ (Equations 7 and 8). Black represents the best fit baseline set, red and magenta colored curves represent positive scalar values, and blue and cyan curves represent negative scalar values. Curves are evaluated from a random sub-sample of 500 parameter sets in our posterior distribution. The dark center line of each trajectory is the median, with the 16-85’th percentiles shaded (representing the 69% center values).

Median trajectories and 69 percentile envelopes from 1,000 random parameter sets sampled from the ensemble varied in each of these directions is shown in Figure 4. The first direction *d*_1_=*d*_1_(***α***) (Figure 4b) corresponds to dynamics related to recovery from infection, and is dominated by *δ*, the infected cell decay rate. Slower clearance of infected cells results in prolonged viral load, and more severe symptoms that last longer. Changes along this direction resemble differences between adult and aged mice [31], supporting the biological significance of this direction.

The second direction *d*_2_=*d*_2_(***α***) (Figure 4a) corresponds to onset dynamics which occur in the first two days of infection; virus that quickly reaches a high viral load corresponds to earlier symptoms than with those with a viral load that is slower to peak. Correlation between time of viral and symptoms peak was observed between the different experimental studies [23, 24, 34, 36], as well as between individual volunteers in an H3N2 study [52].

**Figure 5:**
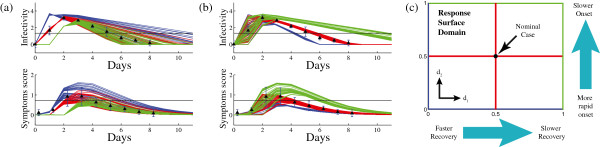
**Variability of host-level response across the response surface.** The colored trajectories in **(a)** and **(b)** correspond to points on the response surface located on equally colored lines shown in **(c)**. The surface is parametrized by the coordinates (*x*_1_,*x*_2_)∈[0,1]×[0,1]. (The point (0.5,0.5) corresponds to the nominal case). The solid black lines represent thresholds above which individuals are assumed to be infectious or symptomatic.

The response surfaces can be represented as arrays (see the Additional file 1) that provide a fast and efficient way to evaluate infection response. Figure 5 shows examples of the computed response surfaces, which are generated by fixing lines along the 4 edges and 2 centerlines of the response surface domain and evaluating Equation 6 (with coefficients taken from tables in Additional file 1: Methods) for points along each fixed line in Figure 5. Position on the response surface is described by coordinates (*x*_1_,*x*_2_) ∈ [ 0,1]× [ 0,1], where the point (0.5,0.5) corresponds to the nominal case (origin of the hyper-plane) associated with the data fitting. Along each line, we fix one scalar value *x*_*j*_,*j*=1,2 and select the other scalar value randomly from a uniform distribution. We repeat this for 35 random trajectories on each line, at *x*_*j*_=0 (blue), *x*_*j*_=0.5 (red) and *x*_*j*_=1.0 (green) for *j*=1 (Figure 5a) and *j*=2 (Figure 5b).

### Within-host epidemiological parameters derived in a population-level model

As a starting point for the full population-scale model, we assign two uniform random numbers (*x*_*j*_∈*U*(0,1),*j*=1,2) to every individual in the population that becomes infected. These numbers correspond to coordinates in the response plane and are used to compute trajectories describing infectivity and symptomaticity of that agent during infection. We assume infection is symptomatic only if the respective trajectory is above a scalar threshold value, which also provide a metric for estimating the duration of latent and incubation periods in the model. The threshold values can be varied according to the particular purpose of the model, as we illustrate in the age-severity study below. Sample thresholds are plotted in Figure 5 as solid black lines.

Symptoms are assigned values 0 (asymptomatic) if below the threshold value or 1 (symptomatic) if above. The symptoms threshold *S*^∗^=0.717 is selected so that 33% of individuals fall below it in order to maintain a percentage of asymptomatic cases consistent with literature estimates [37]. The viral load threshold is selected at *V*^∗^=1.35, which gives a mean duration of infectivity of 5.2 days. The mean onset to infectivity calculated from evaluating the response surfaces is 0.83 days. This threshold was selected so the model would be comparable with estimates in the literature, and a comparison is found in the Discussion [6, 30, 43, 53–59].

**Figure 6:**
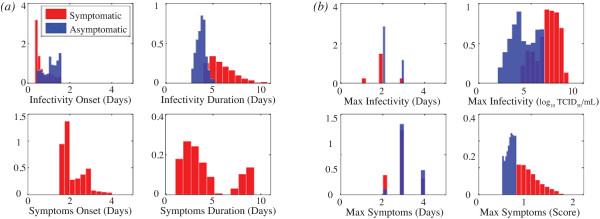
**Histograms showing distribution of onset, duration, and peak of infectivity and symptoms.** Histograms represent distributions across 1 million random numbers (representing infected individuals), evaluated on the response surface (Equation 6). Asymptomatic individuals (maximum symptoms score < 0.717) are shown in blue, while symptomatic individuals are in red. **(a)** Onset and duration of infectivity and symptoms are computed from each response surface evaluation. **(b)** Time and value of infectivity and symptoms at their peak.

The length of onset and duration periods are estimated by interpolating where the evaluated trajectories crosses the threshold line and the means are reported in Table 2. Histograms representing the distributions of times of onset and duration of infectiousness and symptoms, as well as time of peak symptoms and infectivity and corresponding values are shown in Figure 6 for response surface evaluations corresponding to one million random numbers (infected individuals). We separate the asymptomatic cases (maximum symptoms score < 0.717) and show them in red, while symptomatic cases are in blue. The maximum infectivity over all infections is 3.71 (compared to the maximum infectivity data point of 2.88 log10 TCID _50_), which we use as a linear coefficient to normalize computed viral load into transmission probability in the agent-based simulation. We refer to the above described within-host model as the “baseline” model, and calibration of the baseline case is discussed in the Additional file 1.

**Table 2:** **Estimates of epidemiological parameters across response surfaces**

	Total	Symptomatic	Asymptomatic
	(st.d.)	(st.d.)	(st.d.)
Latent period (days)	0.83 (0.40)	0.69 (0.35)	1.11 (0.34)
Incubation period (days)	-	2.14 (0.53)	None
Duration of infectivity (days)	5.17 (1.54)	5.87 (1.38)	3.71 (0.47)
Duration of symptoms (days)	4.18 (2.32)	4.18 (2.32)	None
Peak infectivity time (days)	2.10 (0.51)	2.00 (0.51)	2.29 (0.45)
Peak infectivity value	3.15 (0.31)	3.30 (0.22)	2.85 (0.23)
Peak symptoms time (days)	3.04 (0.58)	2.97 (0.60)	3.19 (0.51)
Peak symptoms score	0.86 (0.25)	0.99 (0.20)	0.60 (0.075)

We estimate epidemiological parameters pertaining to duration and intensity of infectivity and symptoms by taking the average over 1 million randomly generated individuals, and assume all individuals have equal probability of same response to infection. We compute the average times for onset and resolution of infectivity and symptoms by computing when the trajectory crosses the thresholds S* and I* using linear interpolation, using symptoms threshold of S* = 0.717 and viral threshold of I* = 1.35.

### Comparison of the baseline model with a reference ABM within-host model

We first show that the baseline within-host model produces similar results to a standard within-host model used in ABM studies, which we call the “reference” ABM model [4, 10]. In the reference within-host model in FRED, the mean duration of infectivity (4.1 days) and mean latent period (1.2 days) can be evaluated directly using the discrete probability distributions explicitly used in the model, and give an average total duration of infection as 5.3 days from inoculation to resolution [4]. Each infected agent is either symptomatic or not, based on a Bernoulli random variable.

In both cases in this comparison, all individuals who become infectious have an equal probability of contracting the same response, meaning that when an individual becomes infected they are assigned random numbers to represent illness independent of information such as a demographic or pre-existing conditions. Therefore the observed epidemiological differences are due only to the differences in the models of within-host response. The main difference between the two within-host models is that symptomatic individuals have a higher infectivity lasting over a shorter period and asymptomatic individuals have a lower infectivity lasting over a longer period in the reference ABM within-host model, compared to the new baseline within-host model.

To measure the differences resulting from the two within-host models in a population-level simulation, we run each epidemic scenario with 50 distinct random seeds, and compare epidemiological measurements in Table 3. We observe that the peak incidence corresponding to the baseline within-host model occurs earlier than the reference model (31.1 days compared to 25.1 days), which can be attributed to the shorter incubation period and reduced transmission rates in symptomatic individuals. Also observed is a larger peak incidence in the baseline case (36,811 compared to 41,883 infected), attributed to longer duration of infection. The reproductive rate *R*_0_ is estimated by the average number of secondary infections from the original 100 exposed individuals on day 0 computed on day 10. We observe that *R*_0_ is slightly higher in the baseline at 2.14+0.38 than default FRED at 1.98+0.37. We measure the average time to run one simulation over 100 days, and find that the Baseline case has an average run time of 79 seconds and in the standard ABM model has an average run time of 80 seconds on a laptop.

**Table 3:** **Comparison of epidemiological parameters between reference ABM model and baseline**

	Reference ABM (st.d.)	Baseline (st.d.)
Attack rate (AR)	49.6588 (0.105)	50.024 (0.109)
Symptomatic AR	33.271 (0.0879)	33.6062 (0.083)
% Symptomatic	67.00%	67.18%
*R*_0_	2.14 (0.38)	1.98 (0.37)
Peak incidence (Days)	31.12	25.10
Peak incidence (# People)	36,811	41,883
Average run time	80 seconds	79 seconds

Computed parameters are presented as averages (standard deviations) from 50 population-level simulations of influenza epidemics for the 'Reference” FRED within-host model and the “Baseline” within-host model proposed in this manuscript.

### Age-severity study

Aging alters both the innate and adaptive branches of the immune system, influencing response to influenza virus infection [60]. We assume that age of an infected individual maps to a scalar input value along *d*_1_, reflecting longer and more severe infection and symptoms in older individuals. To illustrate the flexibility of our immune-epidemiological approach, we conduct a study where we compare the simulated epidemics between three different age-severity maps: a linear increasing function, and two functions based on mortality data from past epidemics in 1918 and 1957 [61]. Mortality patterns associated with epidemics traditionally form a “U” curve, reflecting higher mortality and more severe disease in the very young and the elderly. A distinctive feature of the 1918-19 influenza pandemic was a “W” shaped pattern in the mortality curve, showing a secondary peak in mortality rate for the 25-35 years age group [62, 63]. The age-severity model thus assumes mortality rates are indicators of severity of disease experienced by different age groups. Piece-wise linear functions map age to [0,1] for all three maps, and mortality data is scaled so the area under all three curves (for *age*∈[0,100]) is preserved. In this way the model considers two factors linking age with disease severity: deterioration of the immune system with age and immunological memory. Immunological memory refers to the adaptive immune response to pathogen the body has previously encountered, and contributes to explaining the “W” shape in the 1918 pandemic mortality curve [64].

**Figure 7:**
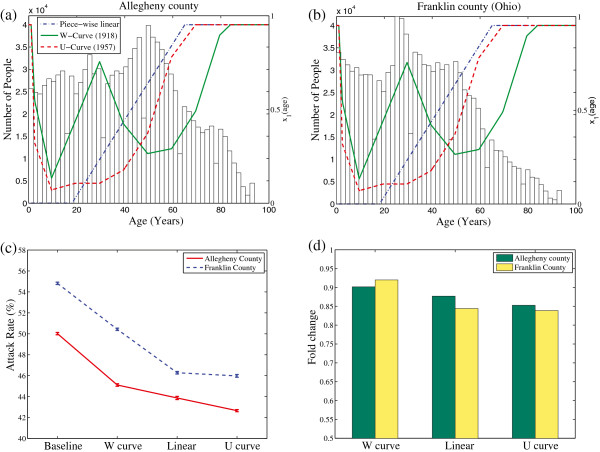
**Age-severity study results.** Panels **(a)** and **(b)** show age-distribution of population in two counties (left axis). Superimposed are three age-severity models showing the assigned value of *x*_1_ coordinate as a function of age. **(c)** Attack rates for each scenario. **(d)** Fold change from the baseline model for each age-severity model.

The median age in the United States is 37.2 years, while Allegheny County has a median age of 41.3 [65], among the oldest in the nation. Franklin County, Ohio is comparable to Allegheny in population size, density and household size (see Table 4), but has a younger age distribution (median age 33.4). The age-severity model is also studied on Franklin County in order to compare how different age distributions affect the overall epidemic. Plots of the three “age-to-phenotype” maps superimposed over age distribution histograms for both counties are presented Figure 7(a)-(b).

**Table 4:** **Summary of demographics**

	Allegheny County	Franklin County
Population size	1,164,879	1,069,386
Land area (miles ^2^)	730.07	532.19
Median age	41.3	33.4
Average household size	2.23	2.38

Comparison of demographic values between Allegheny County, Pennsylvania and Franklin County, Ohio. Source: US Census Bureau [66].

Epidemiological estimates averaged across 50 random simulations are reported in Table 5. In the baseline scenario, the higher attack rate in Franklin County illustrates the role age distribution has on the outcome of an epidemic (Figure 7(c)). Younger individuals, particularly school aged, have higher contact probabilities than older individuals. This is supported by decreased attack rates in both counties when the age-severity models are imposed. Across the three age-severity models, the W-curve severity model has the highest attack rate, illustrating how more severe and prolonged illness in the 25-35 age group can increase epidemic severity compared to either the linear or U-shaped model. The differences between the linear and the U-shaped models are less dramatic, particularly in Allegheny County.

**Table 5:** **Attack rates, reproductive number** ***R***_**0**_ **, and percent symptomatic for Allegheny and Franklin Counties**

	Baseline	Linear	'U’ curve	'W’ curve
AR: Allegheny County (PA)	50.02 (0.11)	43.87 (0.15)	42.66 (0.10)	45.11 (0.12)
AR: Franklin County (OH)	54.84 (0.11)	46.28 (0.12)	45.98 (0.13)	40.43 (0.11)
*R*_0_: Allegheny County (PA)	2.14 (0.38)	1.92 (0.32)	1.93 (0.28)	2.00 (0.33)
*R*_0_: Franklin County (OH)	2.28 (0.34)	2.05 (0.29)	2.05 (0.27)	2.16 (0.30)
%S: Allegheny County (PA)	67%	54.23%	49.25%	62.16%
%S: Franklin County (OH)	67%	48.21%	44.69%	63.16%

Comparison of simulated epidemic outcomes between the baseline model and three age-severity models. All averages and standard deviations are computed over 50 random simulations.

We plot the fold difference in the attack rates in the age-severity models to their baselines in order to make a direct comparison between the models (Figure 7(d)). We observe a larger fold change for the W-model in Allegheny County, showing that for these counties, the decreased illness severity in the 45-65 ranges has a larger impact than the increased illness severity in the 25-35 age range. The larger fold change in the linear and U-shaped models of Franklin County implies a more dramatic change in epidemic outcome when age-severity assumptions are made in the younger age distribution scenario. The model assumes symptomatic cases by determining threshold maximum symptoms score. Less severe illness is more likely to correspond to asymptomatic cases, so the age-severity model also has tendencies to have an age dependence on symptomatic cases. We report the percentage of symptomatic patients in each scenario in Table 5.

## Discussion

The strategy for incorporating the host-level model described here could be employed with other deterministic population level models as well as other well-established ABM simulation platforms such as EpiSimS [3]. Our aim here is to develop a methodology to bridge the gap between within-host models of virus/symptoms dynamics and population-level models that may also capture human movement and demographics. FRED provides a ready to use framework for population-level modeling using the synthetic population data set, allowing easy extension of the within-host model to simulations of populations with different demographic descriptions (here by distribution of age). We note that integration of our within-host model into a deterministic population level model may have similar results with less complexity, and we provide the coefficients for the response surfaces in the supplemental for straightforward use.

Our model implicitly uses age-structured contact patterns in the synthetic data set and ABM, and the results from the age-severity study reflect this relationship between distribution of age and epidemic severity. Previous studies have derived location specific contact matrices, either through use of synthetic populations with ABM network structures [67, 68], or from surveys of European communities [69]. In these cases, two mixing patterns emerge: a strong contact structure between people of similar age and a weaker structure between children and middle-aged adults, most likely due to parent-child contact. Use of contact matrices based on social networks better capture age-specific infection patterns of infectious diseases which helps in estimation of the basic reproductive number *R*_0_. In our age-severity study, we provide a method to further fine-tune these estimates depending on host-response to a circulating strain. The computed values of *R*_0_ in this study are derived from assumptions about one particular experimental strain, and results may vary depending on characteristics unique to different circulating strains.

When integrating our derived within-host model into the population-level framework of FRED, further assumptions were required on scaling and thresholds. The symptoms threshold was set so approximately 1/3 of individuals in the baseline scenario are asymptomatic, consistent with accepted public health estimates [37]. However, for the infectivity threshold *I*^∗^, there was flexibility. Different values of *I*^∗^, correspond to different latent periods, impacting the duration of time an individual is in the exposed category. The threshold (1.35) was selected such that the average baseline latent periods are 0.83 days, and mean duration of infectivity is 5.17 days. Estimates for latent period are experimentally unknown due to difficulty in measuring, but a mathematical model predicts 0.4-1.5 days [53]. Duration of infectivity has been estimated at 4.8 days [37], and various studies have reported estimates at 3.38 days [30], 6.6 days [54], 5.0 days [55], and 3.1 days [43]. Our mean value of 4.98 days thus is consistent with existing estimates. This assumption reflects that in the baseline model, the incubation period exceeds the latent period, so there is a period in which an individual is not symptomatic and shedding virus. In one modeling study, a lag of 1.9 days between latent and incubation periods was assumed [6].

In our study, the incubation period is estimated at 2.14 days, duration of symptoms about 4 days for the symptomatic cases, in which both estimates rely on our choice of symptoms threshold. Incubation period has been estimated anywhere from 1.4 days (1.3 - 1.5 95% CI) [56], 1.5-2 days [57], 2-3 days [58], to 4 days [30]. Estimates for duration of symptoms vary from 4.5-5 days [37], 6 days [59], 5.6 days [55]. We remark that lowering the symptoms threshold would coincide with a shorter incubation period. One estimate that is well agreed upon is that viral load peaks at around 2 days post inoculation, and symptoms peak at around 3 days p.i. [37], which is consistent with our simulation measurements as well.

We selected the mathematical model of Baccam *et al.* [38] to describe viral and symptoms dynamics for three reasons. First, structural identifiability analysis showed parameter values could be estimated given the data. Second, it is the simplest model that generated the required inputs for a population level simulation (infectivity and symptoms score), and last, the model fit the data. Our method also provides a method for varying host-level responses from experimental baseline measurements in order to predict various illness scenarios under different host assumptions. As a starting point, we used a nominal response calibrated to one non-pandemic seasonal human influenza strain (A/Texas/36/91). As more data allows more subtle description of disease phenotypes characterized by more complex within-host models, our method could be easily scaled to such models as, although computation of the response-surfaces is more complex, it is done a priori and thus requires no additional resources for the population-level simulation.

Community based studies also give insight corresponding to our assumption that infection in the elderly may be characterized by longer viral load, and longer and higher symptoms scores. One study suggests a median duration of symptoms of 7 days for the entire community, compared to 8 days for older people [30]. Another study estimates that duration of infectiousness/viral shedding is 6.3 days for children, and 6.7 days for adults [54]. Using a linear mapping from age to infection severity is a simplistic assumption without biological motivation, which is why three different mappings were studied.

Our model estimates of duration and intensity of infectivity and symptoms show asymptomatic infections correspond to reduced viral load, which is consistent with previous studies of asymptomatic disease and transmission, but the degree is unknown [70]. In a community-based study of 2009 H1N1 in Hong Kong, peak viral load averaged over the asymptomatic subjects was 3.2e3 copies/mL compared to 3.6e7 copies/mL averaged over the symptomatic subjects [57]. In a recent human volunteer study of H3N2/Wisconsin, 50% of asymptomatic subjects had evident viral shedding and a reported a significantly reduced viral load in the asymptomatic cases [52]. However, the exact mechanism contributing to asymptomatic infection is not incorporated in this model, and a study of these mechanisms and how they influence an epidemic are important future considerations.

This study makes the assumption that infectivity scales linearly with contact rate and is proportional to the log10 of the viral load as measured by nasopharyngeal swabs. Although this is plausible, the exact relationship between viral load and infectivity on disease transmission is unknown. One model, which studies the relationship between virus/host dynamics and a population-level model, multiplied viral load by the amount of nasal discharge as the estimate of infectivity [71]. Another model of measles used the area under the infected cell curve to model transmission strength by 'amount’ of infection which was also used as a determinant for symptomatic or asymptomatic infection [72]. In a household study of influenza transmission in Hong Kong, models of molecular viral shedding and log10 molecular viral shedding were studied to compare viral load with transmission [57]. The case in which viral load is used as an indicator of transmission leads to nearly all transmission occurring in the first 1-2 days after inoculation, implying that intervention strategies must be rapid. Using the log10 model of viral shedding to transmission corresponds to significant transmission lasting strongly up to 3-4 days after viral inoculation. Therefore, knowing the exact relationship between infectivity and disease transmission would improve modeling and have significant implication for mitigation strategies.

In the mathematical model describing the host immune response, symptoms scores are generated from the biological mechanisms described by the ODE model, which greatly simplifies complex immune system dynamics. The immune system is composed of innate and adaptive components, which respond to viral infection through inflammation and creation of virus-specific antibodies and effector cells to help resolve the infection [73]. Previous models have been studied within the context of improved understanding of population-level dynamics have incorporated interactions of the innate immune system [53, 74]. Incorporating features such as inflammation and virus-specific antibody production would yield more realistic within-host dynamics, and could explicitly account for factors such as preexisting immunity. Importantly, more complex models could be attempted using response surface methodology at little additional computational cost for large-scale simulations. Further, the ensemble methodology allows the computation of the thickness of the response surface, which would add an additional stochastic element better representing our incomplete knowledge of individual responses.

The added flexibility of a mechanistic within-host to population-level models might be particularly relevant when evaluating the impact of host-level containment measures and additional factors known to demonstrate significant within-host effect depending on immune status. The ability to include this flexibility at minimal computational cost also offers exiting possibilities when simulating new variants, co-infection with bacterial pathogens, for providing a biological basis for morbidity, undoubtedly a major driver of agent behavior and cost of disease, and for mortality related to disease, which is currently lacking in existing population-level simulators.

## Conclusions

We have developed a multi-scale immuno-epidemiological ABM model linking an equation-based within-host model to an agent-based population level model. The key innovation is the use of response surface analysis to simplify the computation of output trajectories of the equation-based within-host model, which makes the immuno-epidemiological model just as efficient and scalable as the underlying population-level ABM. The hybrid ABM model replicates results of simulated epidemics with simpler within-host assumptions such as the reference FRED within-host model, and provides additional flexibility to explore questions relevant to host heterogeneity at no extra computational cost.

## Electronic supplementary material

Additional file 1: **Supplemental information for details in the text.** Includes details about the mathematical model, parameter estimation, data used, response surfaces computed, and calibration of the population-scale models. (PDF 300 KB)

## References

[1] Murillo LN, Murillo MS, Perelson AS (2013). **Towards multiscale modeling of influenza infection**. J Theor Biol.

[2] Keeling MJ, Rohani P (2008). Modeling infectious diseases in humans and animals.

[3] Eubank S, Guclu H, Kumar VA, Marathe MV, Srinivasan A, Toroczkai Z, Wang N (2004). **Modelling disease outbreaks in realistic urban social networks**. Nature.

[4] Grefenstette JJ, Brown ST, Rosenfeld R, DePasse J, Stone NT, Cooley PC, Wheaton WD, Fyshe A, Galloway DD, Sriram A, Guclu H, Abraham T, Burke DS (2013). **FRED (A Framework for Reconstructing Epidemic Dynamics): an open-source software system for modeling infectious diseases and control strategies using census-based populations**. BMC Public Health.

[5] Halloran ME, Ferguson NM, Eubank S, Longini IM, Cummings DA, Lewis B, Xu S, Fraser C, Vullikanti A, Germann TC, Wagener D, Beckman R, Kadau K, Barrett C, Macken CA, Burke DS, Cooley P (2008). **Modeling targeted layered containment of an influenza pandemic in the United States**. Proc Natl Acad Sci.

[6] Longini I, Nizam A, Xu S, Ungchusak K, Hanshaoworakul W, Cummings D, Halloran M (2005). **Containing pandemic influenza at the source**. Science.

[7] Lee B, Brown S, Cooley P, Potter M, Wheaton W, Voorhees R, Stebbins S, Grefenstette J, Zimmer S, Zimmerman R, Assi T-M, Bailey RR, Wagener DK, Burke DS (2010). **Simulating school closure strategies to mitigate an influenza epidemic**. J Public Health Manag Pract: JPHMP.

[8] Brown ST, Tai JH, Bailey RR, Cooley PC, Wheaton WD, Potter MA, Voorhees RE, LeJeune M, Grefenstette JJ, Burke DS, McGlone SM, Lee BY (2011). **Would school closure for the 2009 H1N1 influenza epidemic have been worth the cost?: A computational simulation of Pennsylvania**. BMC Public Health.

[9] Cooley P, Brown S, Cajka J, Chasteen B, Ganapathi L, Grefenstette J, Hollingsworth C, Lee B, Levine B, Wheaton WD, Wagener DK (2011). **The role of subway travel in an influenza epidemic: A New York City simulation**. J Urban Health.

[10] Kumar S, Grefenstette J, Galloway D, Albert S, Burke D (2013). **Policies to reduce influenza in the workplace: impact assessments using an agent-based model**. Am J Public Health.

[11] Lee B, Brown S, Korch G, Cooley P, Zimmerman R, Wheaton W, Zimmer S, Grefenstette J, Bailey R, Assi T-M, Burke DS (2010). **A computer simulation of vaccine prioritization, allocation, and rationing during the 2009 H1N1 influenza pandemic**. Vaccine.

[12] Lee B, Brown S, Bailey R, Zimmerman R, Potter M, McGlone S, Cooley P, Grefenstette J, Zimmer S, Wheaton W, Quinn SC, Voorhees RE, Burke DS (2011). **The benefits to all of ensuring equal and timely access to influenza vaccines in poor communities**. Health Aff.

[13] Hellriegel B (2001). **Immunoepidemiology–bridging the gap between immunology and epidemiology**. Trends Parasitol.

[14] Gilchrist MA, Sasaki A (2002). **Modeling host–parasite coevolution: a nested approach based on mechanistic models**. J Theor Biol.

[15] Mideo N, Alizon S, Day T (2008). **Linking within-and between-host dynamics in the evolutionary epidemiology of infectious diseases**. Trends Ecol Evol.

[16] Gandolfi A, Pugliese A, Sinisgalli C (2014). **Epidemic dynamics and host immune response: a nested approach**. J Math Biol.

[17] Kostova T (2007). **Persistence of viral infections on the population level explained by an immunoepidemiological model**. Math Biosci.

[18] Heffernan J, Keeling MJ (2009). **Implications of vaccination and waning immunity**. Proc R Soc B: Biol Sci.

[19] Martcheva M, Pilyugin SS (2006). **An epidemic model structured by host immunity**. J Biol Syst.

[20] Gilchrist MA, Coombs D (2006). **Evolution of virulence: interdependence, constraints, and selection using nested models**. Theor Popul Biol.

[21] Snider D, Bridges C, Weissman D (2010). **Meeting summary of the workshop 'approaches to better understand human influenza transmission**. Approaches to better understand human influenza transmission. Centers for Disease Control and Prevention (United States of America). 4–5 November 2010; Atlanta, Georgia.

[22] Weinstein RA, Bridges CB, Kuehnert MJ, Hall CB (2003). **Transmission of influenza: implications for control in health care settings**. Clin Infect Dis.

[23] Hayden F, Fritz R, Lobo M, Alvord W, Strober W, Straus S (1998). **Local and systemic cytokine responses during experimental human influenze A virus infection**. J Clin Invest.

[24] Fritz R, Hayden F, Calfee D (1999). **Nasal cytokine and chemokine responses in experimental influenza A virus infection: results of a placebo-controlled trial of intravenous zanamivir treatment**. J Infect Dis.

[25] Julkunen I, Melen K, Nyqvist M, Pirhonen J, Sareneva T, Matikainen S (2001). **Inflammatory responses in influenza A virus infection**. Vaccine.

[26] Eccles R (2005). **Understanding the symptoms of the common cold and influenza**. Lancet Infect Dis.

[27] Smith A, Perelson A (2011). **Influenza A virus infection kinetics: quantitative data and models**. Wiley Interdisciplinary Rev: Syst Biol Med.

[28] Beauchemin C, Handel A (2011). **A review of mathematical models of influenza A infections within a host or cell culture: lessons learned and challenges ahead**. BMC Public Health.

[29] Little JW, Gordon RD, Hall WJ, Roth FK (1979). **Attenuated influenza produced by experimental intranasal inoculation**. J Med Virol.

[30] Tuite A, Greer A, Whelan M, Winter A, Lee B, Yan P, Wu J, Moghadas S, Buckeridge D, Pourbohloul B, Fisman DN (2010). **Estimated epidemiologic parameters and morbidity associated with pandemic H1N1 influenza**. Can Med Assoc J.

[31] Toapanta F, Ross TM (2009). **Impaired immune responses in the lungs of aged mice following influenza infection**. Respir Res.

[32] Wheaton W (2012). **2005-2009 U.S. Synthetic Population Ver. 2. RTI International**.

[33] Cajka J, Cooley P, Wheaton W (2010). **Attribute assignment to a synthetic population in support of agent-based disease modeling**. Methods Rep (RTI Press).

[34] Hayden F, Treanor J, Betts RF, Lobo M, Esinhart JD, Hussey EK (1996). **Safety and efficacy of the neuraminidase inhibitor GG167 in experimental human influenza**. J Am Med Ass.

[35] Barroso L, Treanor J, Gubareva L, Hayden F (2005). **Efficacy and tolerability of the oral neuraminidase inhibitor peramivir in experimental human influenza: randomized, controlled trials for prophylaxis and treatment**. Antivir Ther.

[36] Murphy A, Platts-Mills T, Lobo M, Hayden F (1998). **Respiratory nitric oxide levels in experimental human influenza**. Chest.

[37] Carrat F, Vergu E, Ferguson N, Lemaitre M, Cauchemex S, Leach S, Valleron A (2008). **Time lines of infection and disease in human influenza: a review of volunteer challenge studies**. Am J Epidemiol.

[38] Baccam P, Beauchemin C, Macken C, Hayden F, Perelson A (2006). **Kinetics of influenza A virus infection in humans**. J Virol.

[39] La Gruta NL, Kedzierska K, Stambas J, Doherty PC (2007). **A question of self-preservation: immunopathology in influenza virus infection**. Immunol Cell Biol.

[40] Conti B, Taberean I, Andrei C, Bartfai T (2004). **Cytokines and fever**. Front Biosci.

[41] Descotes J, Vial T (2007). Flu-like syndrome and cytokines. Cytokines in Human Health.

[42] Cohen S, Doyle WJ, Skoner DP (1999). **Psychological stress, cytokine production, and severity of upper respiratory illness**. Psychosom Med.

[43] Skoner DP, Gentile DA, Patel A, Doyle WJ (1999). **Evidence for cytokine mediation of disease expression in adults experimentally infected with influenza A virus**. J Infect Dis.

[44] Gentile D, Doyle W, Whiteside T, Fireman P, Hayden FG, Skoner D (1998). **Increased interleukin-6 levels in nasal lavage samples following experimental influenza A virus infection**. Clin Diagn Lab Immunol.

[45] Metropolis N, Rosenbluth A, Rosenbluth M, Teller A, Teller E (1953). **Equations of state calculations by fast computing machines**. J Chem Phys.

[46] Hastings W (1970). **Monte Carlo sampling methods using Markov chains and their applications**. Biometrika.

[47] Gammerman D, Lopas H (2006). Markov chain Monte Carlo stochastic simulation for Bayesian inference.

[48] Earl D, Deem M (2005). **Parallel tempering: theory, applictions and new perspectives**. Phys Chem Chem Phys.

[49] Brown K, Sethna J (2003). **Statistical mechanical approaches to models with many poorly known parameters**. Phys Rev E.

[50] Gutenkunst R, Waterfall J, Casey F, Brown K, Myers C, Sethna J (2007). **Universally sloppy parameter sensitivities in systems biology models**. PLoS Comput Biol.

[51] Lee H, Topham D, Park S, Hollenbaugh J, Treanor J, Mosmann T, Jin X, Ward B, Miao H, Holden-Wiltse J, Perelson A, Zand M, Wu H (2009). **Simulation and prediction of the adaptive immune response to Influenza A virus infection**. J Virol.

[52] Huang Y, Zaas A, Rao A, Dobigeon N, Woolf P, Velman T, ien NO, McClain M, Varkey J, Nicholson B, Carin L, Kingsmore S, Woods C, Ginsburg G, III AH (2011). **Temporal dynamics of host molecular responses to differentiate symptomatic and asymptomatic influenza A infection**. PLoS Genet.

[53] Canini L, Carrat F (2011). **Population modeling of influenza A/H1N1 virus kinetics and symptom dynamics**. J Virol.

[54] Seuss T, Buchholz U, Dupke S, Grunow R, an der Heiden M, Heider A, Biere B, Schweiger B, Haas W, Krause G (2009). **Shedding and transmission of novel influenza virus A/H1N1 infection in households - Germany, 2009**. Am J Epidemiol.

[55] Witkop C, Duffy M, Macias E, Gibbons T, Escobar J, Burwell K, Knight K (2010). **Novel influenza A (H1N1) outbreak at the US Air Force Academy: epidemiology and viral shedding duration**. Am J Prev Med.

[56] Lessler J, Reich N, Brookmeyer R, Perl T, Nelson K, Cummings D (2009). **Incubation periods of acute respiratory viral infections: a systematic review**. Lancet Infect Dis.

[57] Lau L, Cowling B, Chan KH, Lau E, Lipsitch M, Cheng C, Houck P, Uyeki T, Peiris J, Leung G (2010). **Viral shedding and clinical illness in naturally acquired Influenza A virus infections**. J Infect Dis.

[58] Yamagishi T, Matsui T, Nakamura N, Oyama T, Taniguchi K, Aoki T, Hirakawa K, Okabe N (2010). **Onset and duration of symptoms and timing of disease transmission of 2009 influenza A (H1N1) in an outbreak in Fukuoka, Japan, June 2009**. Jpn J Infect Dis.

[59] Lessler J, Reich N, Cummings D (2009). **Outbreak of 2009 pandemic influenza A (H1N1) at a New York City school**. N Engl J Med.

[60] Lambert ND, Ovsyannikova IG, Pankratz VS, Jacobson RM, Poland GA (2012). **Understanding the immune response to seasonal influenza vaccination in older adults: a systems biology approach**. Expert Rev Vaccines.

[61] Luk J, Gross P, Thompson WW (2001). **Observations on mortality during the 1918 influenza pandemic**. Clin Infect Dis.

[62] Dauer C, Serfling R (1961). **Mortality from influenza, 1957–1958 and 1959–1960**. Am Rev Respir Dis.

[63] Palese P (2004). **Influenza: old and new threats**. Nat Med.

[64] Ahmed R, Oldstone MB, Palese P (2007). **Protective immunity and susceptibility to infectious diseases: lessons from the 1918 influenza pandemic**. Nat Immunol.

[65] Howden LM, Meyer JA (2011). Age and sex composition: 2010:2010 census briefs.

[66] Bureau, USCensus (2013). **American fact finder**. Website.

[67] Del Valle SY, Hyman J, Hethcote HW, Eubank SG (2007). **Mixing patterns between age groups in social networks**. Social Netw.

[68] Fumanelli L, Ajelli M, Manfredi P, Vespignani A, Merler S (2012). **Inferring the structure of social contacts from demographic data in the analysis of infectious diseases spread**. PLoS Comput Biol.

[69] Mossong J, Hens N, Jit M, Beutels P, Auranen K, Mikolajczyk R, Massari M, Salmaso S, Tomba GS, Wallinga J, Heijne J, Sadkowska-Todys M, Rosinka M, Edmunds WJ (2008). **Social contacts and mixing patterns relevant to the spread of infectious diseases**. PLoS Med.

[70] Patrozou E, Mermel LA (2009). **Does influenza transmission occur from asymptomatic infection or prior to symptom onset?**. Public Health Rep.

[71] Handel A, Longini IM Jr, Antia R (2007). **Neuraminidase inhibitor resistance in influenza: assessing the danger of its generation and spread**. PLoS Comput Biol.

[72] Heffernan J, Keeling MJ (2008). **An in-host model of acute infection: Measles as a case study**. Theor Popul Biol.

[73] Tamura S, Kurata T (2004). **Defense mechanisms against Influenza virus infection in the respiratory tract mucosa**. Jpn J Infect Dis.

[74] Handel A, Longini IM Jr, Antia R (2010). **Towards a quantitative understanding of the within-host dynamics of influenza A infections**. J R Soc Interface.

[CR75] The pre-publication history for this paper can be accessed here:http://www.biomedcentral.com/1471-2458/14/1019/prepub

