# Supplementary material for: A large-scale immuno-epidemiological simulation of influenza A epidemics
Source: BMC Public Health. 2014 Sep 29;14:1019. doi: 10.1186/1471-2458-14-1019 (PMC4194421; doi:10.1186/1471-2458-14-1019)
Supplement: Supplementary file 1 — Additional file 1: Supplemental information for details in the text. Includes details about the mathematical model, parameter estimation, data used, response surfaces computed, and calibration of the population-scale models. (PDF 300 KB) [file 12889_2014_7130_MOESM1_ESM.pdf]

Supplemental Material:

A multi-scale, hybrid simulation of influenza A epidemics with realistic in-host response

Experimental Data

We used viral load and symptoms data from five randomized, double-blind human volunteer studies conducted between 1995 and 1999 for 84 total individuals infected with A/Texas/36/91 (H1N1) [1, 2, 3, 4, 5]. Of these studies, only one [3] (n=17) does not contain symptoms data.

Symptoms in the four studies were measured on a 0-3 scale, from absent (0) to severe (3) for each of the following: muscle aches, fatigue, headache and fever, nasal stuffiness, earache/pressure, runny nose, sore throat, cough, sneezing, breathing difficulty, myalgia, fatigue, headache, feverish feeling, hoarseness and chest discomfort. Two studies [1, 5], split symptoms into 3 categories: systemic, upper and lower respiratory.

It was reported that across all the studies, of the 67 individuals measured for symptoms, 65 experienced at least one symptom. In all studies that measured symptoms, symptom scores were measured twice a day (hour 0 and hour 12 in each day) and reported at the first time point in the day (hour 0). For this reason, we use hour 6 as the time point to describe symptoms.

Table 1: **Viral and symptoms data.** *Weighted averages of viral load and symptoms score data. Weights are given by number of volunteers in each study. The original data may be found in the publications: ([1, 2, 3, 4, 5])*

time (days)	$\log_{10}$ V	std	time (days)	Symptoms	std
0	-	-	0.25	0.1159	0.1548
1	1.6722	0.5175	1.25	0.2541	0.1290
2	3.1871	0.2709	2.25	0.9121	0.4756
4	2.1691	0.0998	3.25	0.9280	0.4393
3	2.8770	0.1790	4.25	0.6350	0.2377
5	1.5307	0.5602	5.25	0.4552	0.2419
6	0.7798	0.2315	6.25	0.3037	0.1381
7	0.4871	0.2111	7.25	0.1558	0.1181
8	0.1756	0.3463	8.25	0.0899	0.1737

Data abstraction

The viral titers across the five studies ([1, 2, 3, 4, 5]) are all reported in $log_{10}TCID_{50}/mL$, and as averages across individuals in the respective studies. Plots of viral load curves show

that qualitatively they look similar except for variations in the viral magnitude between studies. Mapping viral load to a measure of infectivity for disease transmission requires normalization of the viral load, and the important components of the data to capture are when the viral load increases, peaks, and decreases. To adjust the data, we use mixed-effects modeling to compute a vertical shift for each data curve (random-effect), assuming that the distance between each curve is minimized. For each study, we get the shifts in table 2. We adjusted each curve by the shift and computed weighted means and standard deviations (fixed-effect) on the shifted data. The mean of the data is the same with and without the shifts, but the standard deviation is smaller with the shifts and reflects variability between each study from one mean trajectory.

Table 2: **Mixed-effects of viral load data.** *To average data across five volunteer studies, we used mixed-effects modeling on viral load data to minimize vertical shifts in data curves from each study. The random-effect (shifts) are presented below, and the fixed-effects (mean and standard deviations) are presented in Table 1. We weigh the means by the number of volunteers in each study.*

Study	Hayden98	Fritz99	Barroso05	Murphy98	Hayden96
Reference	[1]	[5]	[3]	[4]	[2]
N	19	8	17	14	26
Random effect	0.4122	-0.1447	-0.3558	0.4108	-0.3225

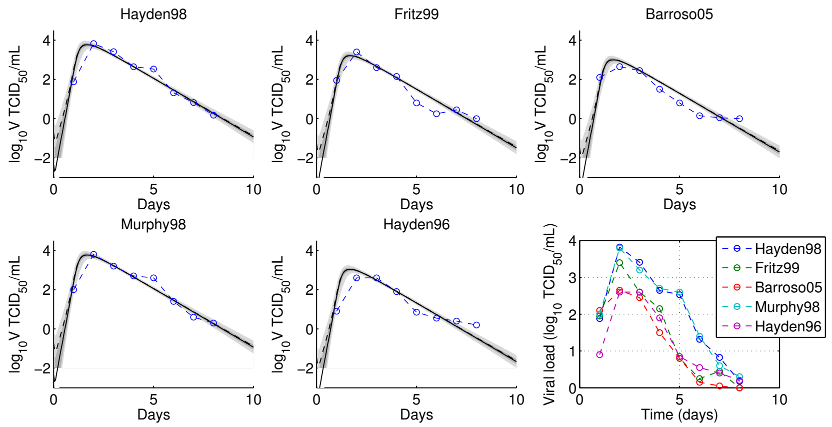

Ensemble model trajectories (see Figure 3 in main text) for viral load with each curve re-adjusted by shift to compare with the original data from the 5 studies: [1, 2, 3, 4, 5]

Parameter Estimation

We write the ODE model for short as $\dot{\mathbf{x}} = \mathbf{f}(\mathbf{x}, \boldsymbol{\alpha})$, in which $\mathbf{x}$ denotes the vector of state variables, and $\boldsymbol{\alpha} = (\alpha_1, \alpha_2, ..., \alpha_6)$ is the vector of parameters of the system. The observed data is denoted by $D = (\bar{x}_i(t_j), \sigma_{\bar{x}_i(t_j)})$, for response i, at time point j, with standard

deviation σ. Here, 2 is the number of variables we fit data to, and we fit at time points 1 to 8 days (or 1.25 to 8.25 days).

Using the standard assumption that the errors on the data are uncorrelated, random, and sampled from a Gaussian kernel, the likelihood $P(D|\boldsymbol{\alpha})$ is computed from the trajectory $\mathbf{x}(t;\boldsymbol{\alpha})$ of the model as an exponential of the objective value, i.e., as $P(D|\boldsymbol{\alpha}) = \exp(-C(\boldsymbol{\alpha}))$, given by [6]

$$C(\boldsymbol{\alpha}) = \sum_{i=1}^{2}\sum_{j=1}^{8} \frac{|x_i(t_j;\boldsymbol{\alpha}) - \bar{x}_i(t_j)|^2}{2\sigma^2_{x_i(t_j)}}.$$

Optimal parameter values for the target-cell model for A/Texas/36/91 are reported in [7], which we use as baseline except we increase the initial inoculum V_0 from 1e-5 to 0.01. We set upper and lower bounds using biological ranges for parameters c, δ, k, and V_0 [8], and 3.5 log scales around the baseline values for the additional parameters, with the exception of the viral production rate p. Preliminary fits showed values of p exploring outside of the bounds, so we increased the upper bound accordingly.

To sample the Bayesian posterior density $P(\boldsymbol{\alpha}|D)$, we choose to run 4 parallel chains of length 1,000,000. We choose inverse temperature values of $\beta = (1, 0.5, 0.25, 0.125)$, and step size $\epsilon = 0.075/\sqrt{\beta}$. The step size ϵ for each chain is chosen so that each chain will have acceptance ratio (i.e., the ratio of accepted $\boldsymbol{\alpha}^*$ to the total number of proposed $\boldsymbol{\alpha}^*$) approximately equal to 0.23, which has been show to provide optimal convergence speed [9]. Acceptance rates are (0.2475, 0.2479, 0.2546, 0.2797). Average swapping rates, from highest energy down are (0.3558, 0.3529, 0.4117).

Response surfaces

The computed domain for response surface evaluation is $[-0.5, 0.5] \times [-0.5, 0.5]$. We linearly mapped input values in $[0, 1]$ to these intervals to use uniform random numbers as input. These arrays may be evaluated up to 11 days of infection, via:

$$\hat{y} = b_0 + b_1 x_1 + b_2 x_2 + b_3 x_1 x_2 + b_4 x_1^2 + b_5 x_2^2. \tag{1}$$

For viral load we have:

$Varray =$

$$\begin{pmatrix} 1.7957 & -0.3485 & -4.4816 & -0.1895 & -0.1568 & -1.6468 \\ 3.2574 & 0.3007 & -1.1052 & -0.6959 & -0.0630 & -2.0984 \\ 2.7126 & 0.8607 & -0.2395 & -0.4676 & -0.0365 & 0.5058 \\ 2.1846 & 1.4181 & -0.2884 & -0.2562 & -0.1603 & 0.5260 \\ 1.5961 & 1.9964 & -0.3433 & -0.2195 & -0.0835 & 0.5198 \\ 0.9935 & 2.5375 & -0.4165 & -0.2218 & 0.0490 & 0.6078 \\ 0.4070 & 3.0739 & -0.4965 & -0.2629 & 0.0129 & 0.6567 \\ -0.1908 & 3.6330 & -0.5486 & -0.3169 & -0.0335 & 0.8026 \\ -0.8006 & 4.2066 & -0.6178 & -0.3140 & -0.0128 & 0.9302 \\ -1.4123 & 4.7840 & -0.7109 & -0.2540 & 0.0305 & 0.9870 \\ -2.0198 & 5.3592 & -0.8238 & -0.1551 & 0.0694 & 0.9632 \end{pmatrix}$$

and for symptoms:
$Sarray =$

$$\begin{pmatrix} 0.0028 & -0.0219 & -0.1371 & 0.0805 & 0.0065 & 0.3258 \\ 0.6238 & 0.2043 & -1.1329 & -0.6542 & 0.0141 & -0.4606 \\ 0.8332 & 0.6492 & -0.5557 & -0.7593 & 0.3423 & -0.3726 \\ 0.6610 & 0.8267 & -0.2723 & -0.5517 & 0.6364 & 0.2271 \\ 0.4482 & 0.7849 & -0.1936 & -0.2763 & 0.7802 & 0.2816 \\ 0.2708 & 0.6001 & -0.1848 & -0.2685 & 0.7596 & 0.2400 \\ 0.1590 & 0.4117 & -0.1449 & -0.2353 & 0.5750 & 0.2031 \\ 0.0922 & 0.2702 & -0.1086 & -0.1761 & 0.4037 & 0.1516 \\ 0.0533 & 0.1730 & -0.0806 & -0.1327 & 0.2722 & 0.1053 \\ 0.0311 & 0.1088 & -0.0582 & -0.1003 & 0.1767 & 0.0716 \\ 0.0181 & 0.0680 & -0.0410 & -0.0736 & 0.1127 & 0.0488 \end{pmatrix}$$

.

Calibration of the intra-host model in FRED

FRED is an open source, C^{++} modeling system developed by the University of Pittsburgh Public Health Dynamics Laboratory in collaboration with the Pittsburgh Supercomputing Center and the School of Computer Science at Carnegie Mellon University. In Allegheny County, there are a total of 1,164,879 individuals represented by synthetic computer agents assigned characteristics and behaviors (age, sex, occupation, household etc.) [10]. We use data from the synthetic population database, which is a freely available database based on 2005-2009 U.S. census data [11]. The data includes, for each household, a latitude/longitude coordinate, income, size, and the sex, race, and age of household occupants, representing the distribution of U.S. households. Allegheny County has 524584 households, with 532 schools, 48703 workplaces, 195 group quarters, and 30583 group quarter residents.

Contact rates between individuals have assigned probabilities, which are derived from MIDAS studies from the 1957-58 Asian influenza pandemic: Ferguson *et al.* [12], Longini *et al.* [13], Germann *el al.* [14] and Halloran *et al.* 2008 [15], and are summarized in [16]. Each type of place (Home, School, Work, Neighborhood) is characterized by two sets of scalar parameters: the number of contacts per infectious person per day, and the probability that a contact transmits infection.

The number of contact opportunities per place per day are calibrated to obey a “30-70" rule, which is that 30% of viral transmission occurs in the home, and 70% occurs elsewhere. These numbers are calibrated as an inverse problem, in which contact opportunities is an input parameter, and measured so that output epidemics obey the desired transmission proportions. Our target for a pandemic is a 33% symptomatic attack rate (ARs) with a global attack rate (AR) of 50%. The 33% clinical attack rate for the baseline emerges naturally from our symptoms threshold, and the calibration process is further refined to maintain this ratio.

For the “baseline" within-host model, the transmission rates per person per location per day are 0.21 in the house, 39.9 in the neighborhood, 1.5 at work, and 13.97 in schools. Over 50 runs, this gives an average of 30.01% transmission in the house, 32.96% in the neighborhood, 24.7% in school and 12.31% at work. For the intra-host model found as default in FRED, the calibration process is the same, but for Allegheny County these give different rates per place per day, which are household contacts = 0.20, neighborhood contacts = 42.48, school contacts = 14.32, and workplace contacts = 1.59.

Within-host model in Reference ABM intra-host model

We describe the within-host assumptions in a reference intra-host model used in ABM studies, which we call the “reference" model [17, 18]. The reference FRED model is the within-host model used when FRED is downloaded or used on the website (http://fred.publichealth.pitt.edu/index.php) in the default mode. In the reference within-host model in FRED, the number of days of infectiousness and symptoms are selected from a probability distribution. Each infected agent is either symptomatic or not, based on a Bernoulli random variable. The infectivity of an agent is assigned one of three values: 0 if the agent is uninfected, 0.5 if the agent is infected and asymptomatic, or 1.0 if the agent is infected and symptomatic. An infected agent passes through stages SEIR or SEiR, in which "I" means infectious and symptomatic, and "i" means infectious and asymptomatic. The length of these phases is determined from sampling cumulative distribution functions which are based on continuous distribution functions that approximate an exponential distribution.

The discrete cumulative distribution function for number of days latent (X = days latent) on the space $(0, 1, 2)$ is given by:

$$F(X) = P(X \leq N) = (0, 0.8, 1.0). \tag{2}$$

The discrete cumulative distribution function for number of days symptomatic or asymptomatic (X = days) on the space $(0, 1, ..., 7)$ is given by:

$$F(X) = P(X \leq N) = (0, 0, 0, 0.3, 0.7, 0.9, 1.0). \quad (3)$$

The values for symptomaticity use infectivity and are assigned values 0 or 1. Days incubating are calculated as either the same number as days latent if the individual is symptomatic, or the duration of infectiousness if the individual is asymptomatic. When symptomaticity is 1.0, it dictates an individual's behavior. For the reference ABM model, the mean duration of infectivity (4.1 days) and mean latent period (1.2 days) can be evaluated directly using the discrete probability distributions explicitly used in the model, and give an average total duration of infection as 5.3 days from inoculation to resolution. We estimate the basic reproductive rate (R_0) as the average number of secondary infections by the initial 100 individuals that are exposed at the beginning of the simulation.

Age-severity models

We use 3 models, both mapping $f : age \to [0, 1]$, where $x_1 = f(age)$ is a measure of disease of severity via evaluation of the response surface for direction 1. Here we describe the 3 models.

Linear model

We assume that individuals with age a_1 or younger develop similarly mild infections ($x_1 = 0$) and those aged a_2 or older develop similarly severe infections ($x_1 = 1$):

$$x_1(age) = \begin{cases} 0, & \text{if } age < a_1, \\ \frac{age - a_1}{a_2 - a_1}, & \text{if } a_1 \leq age \leq a_2, \\ 1, & \text{if } age > a_2. \end{cases} \quad (4)$$

We use $a_1 = 18$ and $a_2 = 65$. The value of $x_1 = 0.5$ corresponds to age of 41.5, which is consistent with the median age of 41 years in Allegheny County. For $age \in [0, 100]$ the area under the curve is 58.5.

'U' and 'W' curve models

We use mortality data published in Luk *et al.* [19] to obtain the shapes of the curves. We assigned each data point to the center point age and connected data points using piece-wise linear interpolation. We set the first and last points (corresponding to < 1 and > 84 years) to a value of 1, corresponding to most severe disease, and scaled the interior data points so that the area under the curves would be 58.5.

For the 'W' curve, corresponding to the mortality from the 1918 pandemic, we used a scale of 1.815/M=7.985e-4, where M is the maximum death toll per 1,000. This gave the normalized values of $(1.0000, 0.5743, 0.1408, 0.4647, 0.7942, 0.4439, 0.2783, 0.3055, 0.5167, 0.9430, 1.0000)$ across the age ranges < 1, $1 - 4$, $5 - 14$, $15 - 24$, ..., $75 - 84$, > 84.

For the ‘U’ curve, we assumed that the last 3 data points are 1, in order to scale in such a way to preserve internal shape. The scale of 0.0186 (18.25/max data point) gave the required AUC. The normalized values are (1.0, 0.3352, 0.0745, 0.1117, 0.1117, 0.1862, 0.3911, 0.8194, 1.0, 1.0 , 1.0)

References

[1] Hayden F, Fritz R, Lobo M, Alvord W, Strober W, Straus S: **Local and systemic cytokine responses during experimental human influenze A virus infection**. *J. Clin. Invest.* 1998, **101**:643–649.

[2] Hayden F, Treanor J, *et al* RB: **Safety and efficacy of the neuraminidase inhibitor GG167 in experimental human influenza**. *J. Am. Med. Ass.* 1996, **275**:295–299.

[3] Barroso L, Treanor J, Gubareva L, Hayden F: **Efficacy and tolerability of the oral neuraminidase inhibitor peramivir in experimental human influenza: randomized, controlled trials for prophylaxis and treatment**. *Antiviral Therapy* 2005, **10**:901–910.

[4] Murphy A, Platts-Mills T, Lobo M, Hayden F: **Respiratory nitric oxide levels in experimental human influenza**. *Chest* 1998, **114**:452–456.

[5] Fritz R, Hayden F, Calfee D: **Nasal cytokine and chemokine responses in experimental influenza A virus infection: results of a placebo-controlled trial of intravenous zanamivir treatment**. *J. Infect. Dis.* 1999, **180**:586–593.

[6] Gammerman D, Lopas H: *Markov chain Monte Carlo stochastic simulation for Bayesian inference*. Boca Raton, FL: Chapman & Hall/CRC, Taylor and Francis Group, 2nd edition 2006.

[7] Smith A, Perelson A: **Influenza A virus infection kinetics: quantitative data and models**. *WIREs Syst. Biol. Med.* 2010, [http://dx.doi.org/10.1002/WSBM.129].

[8] Smith A, Adler F, McAuley J, Gutenkunst R, Ribeiro R, McCullers J, Perelson A: **Effect of 1918 PB1-F2 Expression on Influenza A virus infection kinetics**. *PLoS Computational Biology* 2011, **7**:e1001081.

[9] Chib S, Greenberg E: **Understanding the Metropolis-Hastings algorithm**. *The American Statistician* 1995, **49**(4):327–335.

[10] Cajka J, Cooley P, Wheaton W: **Attribute Assignment to a Synthetic Population in Support of Agent-Based Disease Modeling**. *Methods report (RTI Press)* 2010, **19**(1009):1.

[11] Wheaton W: **2005-2009 U.S. Synthetic Population Ver. 2. RTI International.** 2012. [https://www.epimodels.org/midas/Rpubsyntdata1.do.].

[12] Ferguson N, Cummings D, Cauchemez S, Fraser C, Riley S, Iamsirithaworn AMS, Burke DS: **Strategies for containing an emerging influenza pandemic in Southeast Asia**. *Nature* 2005, **437**:209–214.

[13] Longini I, Nizam A, Xu S, Ungchusak K, Hanshaoworakul W, Cummings D, Halloran M: **Containing pandemic influenza at the source**. *Science* 2005, **309**(5737):1083–1087.

[14] Germann TC, Kadau K, Longini Jr IM, Macken CA: **Mitigation strategies for pandemic influenza in the United States**. *Proceedings of the National Academy of Sciences* 2006, **103**(15):5935–5940.

[15] Halloran ME, Ferguson NM, Eubank S, Longini IM, Cummings DA, Lewis B, Xu S, Fraser C, Vullikanti A, Germann TC, et al.: **Modeling targeted layered containment of an influenza pandemic in the United States**. *Proceedings of the National Academy of Sciences* 2008, **105**(12):4639–4644.

[16] Cooley P, Brown S, Cajka J, Chasteen B, Ganapathi L, Grefenstette J, Hollingsworth C, Lee B, Levine B, Wheaton W, et al.: **The Role of Subway Travel in an Influenza Epidemic: A New York City Simulation**. *Journal of Urban Health* 2011, **88**(5):982–995.

[17] Kumar S, Grefenstette J, Galloway D, Albert S, Burke D: **Policies to Reduce Influenza in the Workplace: Impact Assessments Using an Agent-Based Model**. *American Journal of Public Health* 2013, (0):e1–e6.

[18] Grefenstette JJ, Brown ST, Rosenfeld R, DePasse J, Stone NT, Cooley PC, Wheaton WD, Fyshe A, Galloway DD, Sriram A, et al.: **FRED (A Framework for Reconstructing Epidemic Dynamics): an open-source software system for modeling infectious diseases and control strategies using census-based populations**. *BMC public health* 2013, **13**:940.

[19] Luk J, Gross P, Thompson WW: **Observations on mortality during the 1918 influenza pandemic**. *Clinical Infectious Diseases* 2001, **33**(8):1375–1378.
